# Complete genome sequence dataset of enthomopathogenic *Aspergillus flavus* isolated from a natural infection of the cattle-tick *Rhipicephalus microplus*

**DOI:** 10.1016/j.dib.2023.109053

**Published:** 2023-03-12

**Authors:** Cesar A. Arreguin-Perez, Estefan Miranda-Miranda, Jorge Folch-Mallol, Eduardo Ferrara-Tijera, Raquel Cossio-Bayugar

**Affiliations:** a Centro Nacional de Investigación Disciplinaria en Salud Animal e Inocuidad del INIFAP. Boulevard Cuauhnahuac No. 8534, Jiutepec, Morelos 62574, Mexico; b Centro de Investigaciones en Biotecnología de la Universidad Autónoma del Estado de Morelos Campus Cuernavaca, Universidad Nacional Autónoma de México, Avenida Universidad, 2001, Apartado Postal 510–3, Cuernavaca, Morelos 62210, Mexico; c Servicio Nacional de Sanidad, Inocuidad y Calidad, Secretaria de Agricultura y Desarrollo Rural (SADER). Boulevard Cuauhnahuac No. 8534, Jiutepec, Morelos 62574, Mexico

**Keywords:** *Aspergillus flavus*, Entomopathogen, Biocontrol, Cattle tick, Acaricide

## Abstract

As the most important bovine ectoparasite, the southern cattle tick *Rhipicephalus microplus* transmits lethal cattle diseases such as babesiosis and anaplasmosis, costing the global livestock industry billions of dollars annually. To control cattle ticks, preventive treatment of cattle with pesticides is a common practice; however, after decades of chemical treatment, pesticide resistance has arisen in cattle ticks, rendering most formulations ineffective over time. Facing the perspective of running out of effective chemical treatments against *R. microplus*, research on biocontrol alternatives is necessary. Acaro-pathogenic microorganisms isolated from different developmental stages of *R. microplus* offer potential as biocontrol agents. *Aspergillus flavus* strain INIFAP-2021, isolated from naturally infected cattle ticks, produced high levels of mobility and mortality in the tick population during experimental infections. The whole genome of the fungi was sequenced using the DNBSEQ platform by BGI. The genome was assembled using SOAPaligner, and *A. flavus* NRRL3357 was used as the reference genome; the complete genome contained eight pairs of chromosomes and 36.9 Mb with a GC content of 48.03%, exhibiting 11482 protein-coding genes. The final genome assembly was deposited at GenBank as a bio project under accession number PRJNA758689, and supplementary material is accessible through Mendeley DOI: 10.17632/mt8yxch6mz.1.

**Specifications Table**

**Table:** 

Subject	Biological science

Specific subject area	Genomics. Microbiology
Type of data	Genome sequencing in FASTA format Table Fig.
How the data were acquired	PCR amplification, and phylogenetic inference, genome sequencing was performed using high throughput DNA sequencing using DNBSEQ platform.
Data format	Fasta sequences of complete chromosomes of *A. flavus* in raw, analyzed and filtered formats
Description of data collection	Phylogenetic analysis of an unknown Aspergillus isolate that infected *R. microplus* eggs. Then, genomic DNA was isolated from *Aspergillus flavus* INIFAP-2021. The sequencing libraries were generated using 1μg genomic DNA that was randomly fragmented by Covaris. The fragmented genomic DNA was selected by Agencourt AMPure XP-Medium kit to an average size of 200-400 bp. Fragments were end repaired and then 3’ adenylated. Adaptors were ligated to the ends of these 3’adenylated fragments. This process was used to amplify fragments with adaptors from the previous step. PCR products were purified with the Agencourt AMPure XP-Medium kit. The double stranded PCR products were heat denatured and circularized by the splint oligo sequence. The single strand circle DNA (ssCir DNA) was formatted as the final library. Libraries were sequenced by BGISEQ-500: ssCir DNA molecule formed a DNA nanoball (DNB) containing more than 300 copies through rolling-cycle replication. The DNBs were loaded into the patterned nanoarray by using high-density DNA nanochip technology. Finally, pair-end 100 bp reads were obtained by combinatorial Probe-Anchor Synthesis (cPAS). The genome was assembled using SOAPaligner with *A. flavus* NRRL3357 genome (GCA_014117465.1) as reference genome. The annotation was made with EggNOG *ab initio* as gene finding mode.
Data source location	Institution: CENID-SAI, Instituto Nacional de Investigaciones Forestales Agricolas y Pecuarias City/Town/Region: Jiutepec, Morelos Country: Mexico Latitude and longitude for samples/data collection: 18°53′01.4"N 99°09′27.1"W
Data accessibility	Repository name: GenBank Data identification number: This whole genome project has been deposited at GenBank with accession number ASM1988044v1 Direct URL to data: https://www.ncbi.nlm.nih.gov/assembly/GCA_019880445.1#/def Direct URL to data: All data in this paper is available at NCBI with BioProject number PRJNA758689 Direct URL to data: https://www.ncbi.nlm.nih.gov/bioproject/PRJNA758689/ Genome annotation and amplicon are available from Mendeley dataset Arreguin Perez, Cesar Alejandro (2022), “Data in brief *A. flavus* INIFAP-2021 genome”, Mendeley Data, V1, doi: 10.17632/mt8yxch6mz.1 *ITS* amplicon is also available in GenBank with accession number ON644422 *VBS* amplicon is also available in GenBank with accession number ON716447 *nuSSU* amplicon is also available in GenBank with accession number ON716448 *CMD* amplicon is also available in GenBank with accession number ON716449

## Value of the Data

• The genome data of *Aspergillus flavus* INIFAP-2021 isolated from *R. microplus* infection provides insight into the genetic diversity of *A. flavus* and essential genetic information to reveal important details of its general metabolism.
• These data can be used as initial information for researchers working on fungal microbiology, *Aspergillus*-like organism genetics and biocontrol biotechnology.
• The genome assembly could be used to identify genes involved in *A. flavus* infection of *R. microplus,* particularly those that could act as virulence factors.
• The genome assembly could be used for comparative genome studies to identify differences between *A. flavus* strains capable of infecting different organisms in different environments.
• The genome dataset helps to identify the proteins in metabolic routes involved in the production of mycotoxins that may be the target for manipulation and/or suppression of toxicity of *A. flavus* destined for biocontrol in cattle.
• Our database may be useful for taxonomical classification within the Aspergillus genus

## 1 Objective

The identification of *A. flavus* genes involved in the cattle tick infection may be useful for biocontrol purposes and assessment of mycotoxin-producing potential.

## 2 Data Description

**Fig. 1:**
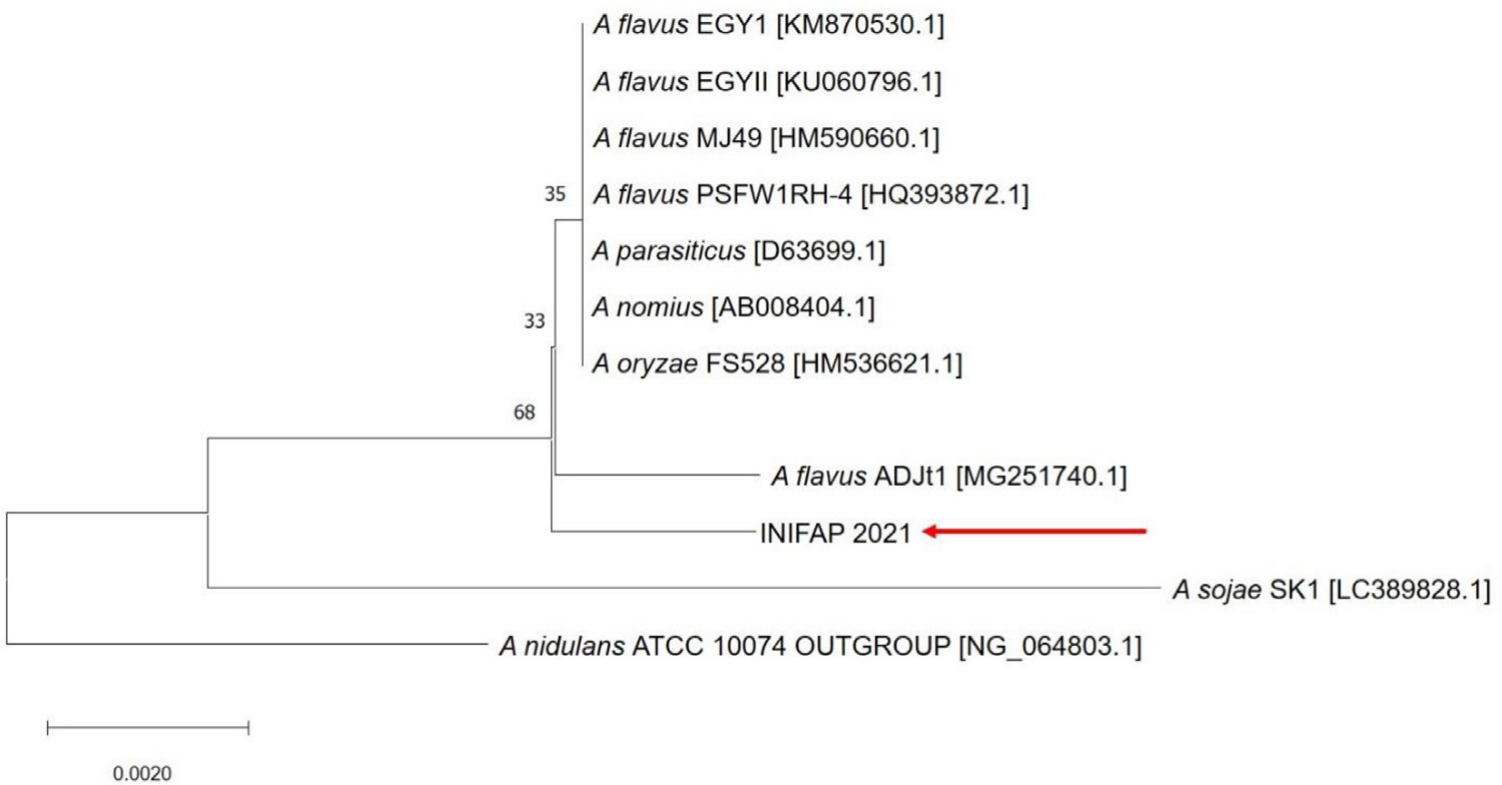
Phylogenetic tree of the *Aspergillus flavus* INIFAP 2021 RNA nuSSU region compared with other *Aspergillus* species and strains. The accession numbers are shown between brackets.

**Fig. 2:**
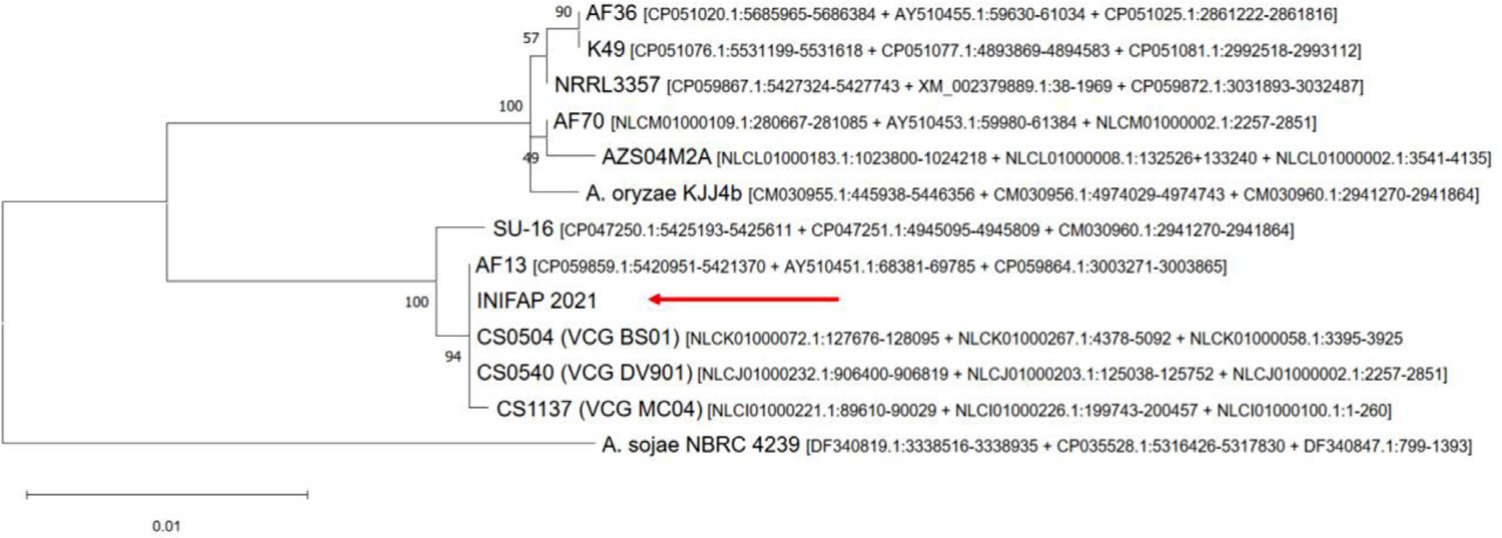
Phylogenetic tree of strain *Aspergillus flavus* INIFAP 2021 calmodulin (*CMD*), versicolorin B sintetase (*VBS*) and internal transcribed spacer region (*ITS*) concatenated genes compared with other *A. flavus* strains and *A. sojae* as an outgroup*.* The accession numbers are shown between brackets.

*A. flavus* is a well-known human and plant pathogen [1], [2], [3], [4], [5] that has been shown to have an effect on the mortality of *R. microplus*. Additionally, some *A. flavus* strains have been shown to produce aflatoxins, which are considered to be carcinogenic compounds, and the corresponding aflatoxin synthesizing genes have been identified [2,6,7]. *A. flavus* has also been reported as a potential biocontrol agent for cattle ticks [8]. Strain *A. flavus* INIFAP 2021 was collected from ill females of *R. microplus* and is now being examined to further characterize its genome. In this work, we first searched the phylogenetic relationship of INIFAP 2021 strain within the Aspergillus genus with nuSSU single phylogeny (Fig. 1) and concatenated phylogeny with calmodulin, versicolorin b sintetase and internal transcribed spacer region markers (Fig. 2).

The sequencing resulted in 8,764,210 reads equivalent to 1,314 raw Mb and 1,273 clean Mb, giving ∼34.4x read depth. The assembly has a total length of 36,944,197 bp distributed in 8 chromosomes with an N50 of 4,658,663. The assembly was uploaded to GenBank with accession number ASM1988044v1. L morphotype genome ranges between 37-37.5 Mpb and S morphotype genomes range between 38.1 and 38.3 Mbp [9], placing this genome in the L morphotype range.

The genome annotation resulted in 11,482 predicted coding sequences (CDS), which were assigned by alignments using databases as follows: 5543 (48.28%) with GO mapping, 2886 (25.14%) with BLAST, 553 (4.82%) with Go-Slim and 2499 (21.76%) with no hits. These predicted genes had a minimum length of 190, a maximum length of 23,271 and an average length of 1604 bp. In addition, another gene prediction was made with EggNOG, resulting in 3791 GO annotated sequences with 8.91 GOs per sequence indicating there are multiple GO numbers for the annotated sequences and supporting the annotation data, this annotated sequences had an average length of 533 bp. Additionally, EggNOG distributed CDS in 4 different COG categories, 1348 (12.02%) for information storage and processing, 1548 (13.8%) for cellular processes and signaling, 3546 (31.61%) for metabolism and 2802 (24.98%) for poorly characterized. Table 1

**Table 1:** Genome features of *Aspergillus flavus* INIFAP-2021.

Genome feature	Value
Genome size	36,994,197
N_50_	4,658,663
Minimum Length	3,032,964
Maximum Length	6,386,555
Average Length	4,624,274
CDS number	11,482

## 3 Experimental Design, Materials and Methods

### 3.1 Fungal strain and DNA extraction

Strain *Aspergillus flavus* INIFAP-2021 was obtained from Arthropodology Laboratory, CENID-SAI, Mexico. This strain was isolated from an *R. microplus* female with a natural fungal infection acquired at the bovine tick-infestation stables and named isolate INIFAP-2021. The isolate spores were cultured in Sabouraud-agar medium for three days at 28°C, and the biomass was collected and dried on filter paper, frozen to -70°C and ground to powder in a mortar while maintaining the freezing conditions according to a previously described procedure [8].

The powder was suspended in lysis buffer (20 mM Tris HCl, 5 mM EDTA, 0.4 M NaCl and 1% SDS, pH 8) and incubated for one hour at 60°C. The DNA from the lysate was extracted with the phenol-chloroform-isoamyl alcohol (25:24:1) technique described previously [10], and the sample was extracted with an equivalent volume of 96% ethanol. The sample was then centrifuged at 13000 rpm for one min, the supernatant was discarded, and the pellet was washed with 70% ethanol and centrifuged once again at 13000 rpm for one min. The liquid was discarded and the pellet was dried and resuspended in 50 µL of TE buffer (10 mM Tris HCl and 1 mM EDTA, pH 8). The DNA concentration was inferred using Nabi by Microdigital.

### 3.2 Species identification

The DNA fragment was amplified using a universal primer set for *nuSSU*: nu-SSU-0817 (5′-TTAGCATGGAATATRRAATAGGA-3′) nu-SSU-1536 (5′-ATTGCAATGCYCTATCCCCA-3′) with an amplicon length of ∼ 720 bp according to previously described methods [8,11]. A primer set for calmodulin (*CMD*): CMD5 (5′-CCGAGTACAAGGARGCCTTC-3′) CMD6 (5′-CCGATRGAGGTCATRACGTGG-3′) [12] with amplicon length ∼ 550 bp and a primer set for versicolorin B sintetase (*VBS*): AFVBSF (5′-AGGCGCATACGATATGTGS-3′) AFVBSR (5′-AACAGACCCTTACGCTGCT-3′) specifically designed for this project with an amplicon length ∼ 717 bp were also used for PCR amplification. The PCR master mix contained 1x PCR buffer, 100 pg of fungal DNA, 20 pmol of corresponding reverse and forward primers, with dNTPs at 2 mM, MgCl_2_ 5 mM and 2 units of Taq polymerase in a total volume of 20 µL. The cycling conditions were 94°C for 5 min followed by 30 cycles of 94°C for 30 s, 55°C 30 s and 72°C for 30 s and a final extension of 72°C for 5 min. Amplicons were verified by 1% agarose electrophoresis in 1x TBE buffer according to a described procedure (Sambrook & Russell, 2001). The amplicons were purified using the Wizard® SV Gel and PCR Clean-Up System (Promega) according to the product instructions and submitted for Sanger commercial sequencing at the “Unidad de Sintesis y Secuenciacion de DNA del Instituto de Biotenologia” UNAM using the same oligonucleotides used for amplification. The obtained sequences of nuSSU were compared using the Basic Local Alignment Search Tool (BLAST) [13] at the NCBI database (BLAST: Basic Local Alignment Search Tool) to obtain similar sequences. Multiple sequence alignment was carried out with Multiple Sequence Comparison by Log-Expectation (MUSCLE) at the EMBL-EBI resources. The nuSSU phylogeny analysis was conducted with MEGA X using the maximum likelihood method with the general time reversible (GTR) model [14] model with a 1000-repeat bootstrap test (Fig. 1).

Phylogenetic analysis of the concatenated calmodulin (*CMD*), versicolorin B synthetase (*VBS*) and internal transcribed spacer (*ITS*) concatenated genes was performed using the maximum likelihood method with a general time reversible (GTR) model [14] with a 1000-repeat bootstrap test (Fig. 2). The *ITS* sequence used in the phylogeny was obtained directly from the genome sequence using BLASTn and the *ITS* sequence from *A. parasiticus* (AY373859.1) was used as a query.

### 3.3 Genome sequencing and assembly

>3 mg of DNA sample was sent to BGI Genomics for further sequencing and assembly. The sequence genome was obtained using the DNBSEQ platform by BGI. The genome was assembled using SOAPaligner with the *A. flavus* NRRL3357 genome (GCA_014117465.1) as a reference genome, and the genome sequence was then uploaded to GenBank (PRJNA758689).

### 3.4 Genome annotation

Genes were predicted in the *Aspergillus flavus* INIFAP 2021 genome based on a generalized hidden Markov model and the gene finding mode as *ab initio* prediction, using the software Augustus within OmicsBox with *Aspergillus oryzae* as the closest species.

The homologues of the proteins of the genome of *Aspergillus flavus* INIFAP 2021 were searched using GO mapping, GO annotation, BLAST and InterProScan.

## Ethics Statements

Neither human nor vertebrate experimentation was conducted, and the ethical committee approved this experiments.

## Data Availability

https://www.ncbi.nlm.nih.gov/assembly/GCA_019880445.1/#/defhttps://www.ncbi.nlm.nih.gov/bioproject/PRJNA758689/.

Genome annotation and amplicon are available from Mendeley dataset Arreguin Perez, Cesar Alejandro (2022), “Data in brief *A. flavus* INIFAP-2021 genome”, Mendeley Data, V1, doi: 10.17632/mt8yxch6mz.1.

*ITS* amplicon is also available in GenBank with accession number ON644422.

*VBS* amplicon is also available in GenBank with accession number ON716447.

*nuSSU* amplicon is also available in GenBank with accession number ON716448.

*CMD* amplicon is also available in GenBank with accession number ON716449.

## Credit Author Statement

**Cesar A. Arreguin-Perez**: Investigation, Formal analysis, Writing – original draft, DNA sample purification PCR, phylogeny; **Estefan Miranda-Miranda** Conceptualization, Sample isolation, PCR workflow determination, DNA samples, Writing – Review & Editing; **Jorge Folch-Mallol**, Data recollection, PCR; **Eduardo Ferrara-Tijera,** Genome annotation; **Raquel Cossio-Bayugar**: Conceptualization, Sample isolation, Writing – Review & Editing, Funding acquisition, Project administration.

## Declaration of Competing Interest

The authors declare that they have no known competing financial interests or personal relationships that could have appeared to influence the work reported in this paper.

## Data Availability

INIFAP-2021 Genome annotation and DNA markers amplicons (Original data) (Mendeley Data).
